# Uneven global coverage of halophilic metagenomes limits comparative analyses of microbial adaptation to saline environments

**DOI:** 10.1007/s42770-026-02017-4

**Published:** 2026-07-22

**Authors:** Camila de Souza Vieira, Leandro Nascimento Lemos, Daniel Kumazawa Morais, Alexandre Soares Rosado, Victor Satler Pylro

**Affiliations:** 1 King Abdullah University of Science and Technology (KAUST), Thuwal, Saudi Arabia; 2 Universidade Federal de Lavras (UFLA), Lavras, Brazil; 3 Ilum School of Science, Brazilian Center for Research in Energy and Materials (CNPEM), Campinas, São Paulo, Brazil; 4 Norwegian College of Fishery Science, UiT, The Arctic University of Norway, Tromsø, Norway

**Keywords:** Hypersaline environments, Metagenomics, Halophilic microorganisms, Metadata curation, Research inequality

## Abstract

Halophilic microorganisms are central to biotechnology, bioremediation, and astrobiology because they persist under extreme and polyextreme conditions analogous to extraterrestrial environments. Although metagenomics has transformed the study of halophilic biodiversity, available datasets remain fragmented and unevenly documented. To assess how halophilic metagenomic research reflects the global exploration of hypersaline environments, we analyzed PubMed-indexed studies and associated sequencing metadata deposited at the National Center for Biotechnology Information (NCBI) Sequence Read Archive (SRA) using a curation workflow. Our quantitative analysis reveals a severe geographic bias linked to uneven global research investment (Gini coefficient = 0.736), with a small number of countries contributing to many publicly available datasets. In contrast, the environmental distribution of these samples showed moderate ecological uniformity (Pielou’s Evenness = 0.818), though we identified pervasive gaps in metadata completeness that hinder dataset interoperability. Our curated dataset highlights a strong research focus on polyextremophilic habitats, positioning these ecosystems as prime targets for biotechnological and astrobiological bioprospecting. Additionally, the geographical bias highlights the need for interoperable global data frameworks and more equitable investment in data generation and analysis, especially in underrepresented regions of the Global South.

## Introduction

Extreme habitats are of particular interest due to the selective conditions they impose, which favor microorganisms with unique adaptations of both biotechnological and astrobiological significance (LoPresto 2024; Mythrayee and Gayathri 2024). While halophilic and halotolerant microorganisms are primarily characterized by their ability to withstand high sodium chloride concentrations, certain taxa can also tolerate a range of concurrent stressors, including extreme temperatures, fluctuating pH levels, and intense ultraviolet radiation. In a biotechnological context, the biosurfactants and enzymes produced by these polyextremophilic representatives retain functionality under multiple harsh conditions, making them highly valuable for industrial processes and bioremediation. Moreover, such polyextremophilic microorganisms serve as excellent analogs in astrobiology, as their environments share remarkable features with extraterrestrial settings, such as Mars and the icy moons Europa and Enceladus (De Souza Vieira and Tótola 2025). Despite their immense potential, characterizing these specialized organisms used to be a challenge, as many require complex, combined parameters for growth that are difficult to replicate in laboratory settings (Xing et al. 2025).

The advent of metagenomics has made it possible to elucidate the taxonomic and functional roles of halophilic and halotolerant communities. The term "metagenome" was introduced in 1998 to describe the collective genomes present in a given environment, reflecting the recognition that most microorganisms cannot be cultivated by traditional methods. The subsequent development of next-generation sequencing (NGS) transformed this concept into a feasible, high-throughput tool for analyzing complex microbial communities (Garrido-Cardenas and Manzano-Agugliaro 2017). Coupled with advances in bioinformatics, these technologies have fundamentally deepened our understanding of microbial life in saline environments. Such habitats are widespread on Earth, spanning saline lakes, brines, salt pans, salt flats, mangroves, and diverse marine ecosystems, each presenting unique combinations of selective environmental conditions (Ben Abdallah et al. 2024; Saccò et al. 2021).

Although significant research efforts have been dedicated to exploring these environments, the current state of knowledge regarding halophilic and halotolerant metagenomes remains fragmented and unevenly distributed. This imbalance imposes a challenge on our ability to draw comprehensive analyses about microbial diversity, ecological patterns, and functional potential across different saline ecosystems. Much of this uneven distribution stems from a profound geographic bias in sampling efforts, as broader trends in microbiome research indicate that the vast majority of samples are collected from Western countries. Consequently, vast global areas are left underrepresented, resulting in a fundamentally biased understanding of these ecosystems (Blake 2024). Furthermore, the fragmentation of current knowledge is exacerbated by inconsistent data management practices. As microbiome science has evolved into a highly data-driven discipline, simply depositing raw sequencing data to meet journal requirements is no longer sufficient; because crucial environmental metadata are frequently omitted, cross-study comparisons become challenging (Huttenhower et al. 2023).

In this study, we hypothesized that the global availability of halophilic metagenomes is driven primarily by regional economic and infrastructural capacities, rather than the true global distribution of saline ecosystems. To test this, we conducted a metadata-driven bibliometric analysis of metagenomic sequences deposited in the National Center for Biotechnology Information (NCBI) Sequence Read Archive (SRA) and linked to PubMed-indexed publications published between 2013 and mid-2025. Specifically, this study aims to: (i) quantitatively assess the global spatiotemporal and environmental distribution of available datasets; (ii) identify structural gaps in metadata reporting that hinder data interoperability; and (iii) evaluate how geographical and infrastructural disparities limit our knowledge of the biodiversity within saline environments.

## Methods

### Dataset selection

The literature search was conducted in the PubMed database using the Entrez Direct (EDirect) package (Kans 2013). To maximize sensitivity and minimize the risk of omitting relevant studies due to the highly fragmented terminology used across fields (e.g., interchangeable use of 'saline', 'hypersaline', 'brine', or 'salt-tolerant'), a broad search string was deployed: “halophilic metagenome” OR “halophiles AND metagenome” OR “halotolerant AND metagenome” OR “hypersaline environment AND metagenome” OR “hypersaline AND metagenome” OR “salt environment AND metagenome” OR “salt soil AND metagenome” OR “salt tolerant AND metagenome” OR “salt lake AND metagenome” OR “salt AND metagenome” OR “saline AND metagenome” OR “saline environment AND metagenome” OR “saline soil AND metagenome” OR “saline lake AND metagenome”. In this study, the term 'halophilic metagenomic' is used operationally to refer to shotgun metagenomic datasets obtained from saline, hypersaline, or salt-affected environmental samples, rather than datasets empirically proven to be taxonomically dominated by obligate halophilic microorganisms.

Although we attempted to automate the retrieval of Sequence Read Archive (SRA) accession numbers directly through programmatic queries, the vast majority of publications did not programmatically link their sequencing data to the article metadata. This technical limitation required a comprehensive manual inspection of each article to identify dataset deposition codes.

To filter the noise generated by our high-sensitivity search string, we established a multi-step manual curation workflow. First, titles and abstracts were screened to exclude off-target, host-associated microbiome studies by discarding records containing the words *gut*, *skin*, *disease*, *bile, host, human, or animal*. Concurrently, studies relying exclusively on marker-gene profiling (16S rRNA or amplicon sequencing) without incorporating shotgun metagenomics were removed. In the second stage, we first performed a careful reading of the remaining abstracts; subsequently, the full texts of the surviving articles underwent a comprehensive evaluation. This final screening step ensured that the retained datasets corresponded exclusively to publicly available shotgun metagenomic data derived from natural saline or hypersaline environments.

The metadata was downloaded using the efetch tool of the EDirect package (Kans 2013). We selected only those articles that included shotgun metagenomic sequences available in the NCBI Sequence Read Archive (SRA) database and associated with BioSamples containing latitude and longitude data.

### Data analysis

All analyses and visualizations were conducted in R (R Core Team 2025) using publicly available packages. Sampling locations were identified from latitude and longitude metadata. The world map with sample points was created using the *maps* package **(**Becker et al. 2025). For the analysis of samples per country, coordinates were converted into geospatial points with the *sf* (Simple Features) package (Pebesma 2018) and overlaid with country shapefiles obtained from *rnaturalearth (*Massicotte and South 2025) and *rnaturalearthdata (*South et al. 2025). This procedure allowed us to assign each sample to a country and produce a map of samples per country. The same country information was combined with NCBI BioSample release dates to generate the temporal distribution of samples per country. We explicitly note that these temporal metrics represent repository availability and database ingestion trends over time, rather than real-time field collection dates.

To standardize the heterogeneous environment metadata across the dataset, samples were classified into unified broad-scale environmental categories by evaluating three core criteria: metadata attribute, hierarchical aggregation, and syntactic normalization. First, assignments were verified by cross-referencing three distinct metadata layers for each record, the submitted broad-scale environmental context, the local-scale environmental context, and the designated metagenomic scientific name (e.g., hypersaline lake metagenome), to ensure ecological consistency. Second, highly granular local descriptions were hierarchically rolled up into overarching environmental classes to prevent sample fragmentation; for instance, localized samples annotated as hypersaline lake metagenome or lake water metagenome were consolidated under the unified Lake category, while varying high-salinity matrices (e.g., salt marsh metagenome) were standardized under Brine. Finally, syntactic and semantic normalization rules were applied programmatically to strip leading/trailing whitespaces, resolve character case discrepancies, and reconcile synonymous environmental descriptions into single string literals for clean downstream grouping. All graphics and maps were produced using the *ggplot2* package (Wickham 2016).

To mathematically quantify sampling inequality, the Gini coefficient was calculated for geographic distribution using the *ineq* package (Zeileis 2014), and Pielou's Evenness index (derived from the Shannon diversity index) was calculated for environmental categories using the *vegan* package (Oksanen et al. 2022).

## Results and discussion

### Dataset selection

The initial PubMed search retrieved 1,259 articles. Due to the high sensitivity of our broad search query, a substantial portion of the initial hits represented off-target disciplines. During the title and abstract screening phase, 425 articles were explicitly excluded for focusing on host-associated microbiomes (e.g., human, animal, or clinical settings), and 214 articles were discarded for exclusively utilizing marker-gene amplicon sequencing rather than shotgun metagenomics. Additionally, 384 articles were excluded following a careful manual reading of their abstracts, as they did not meet the inclusion criteria (e.g., being review articles, isolate genomics studies, or focused on non-saline environments). After this extensive manual curation to remove unrelated and non-metagenomic studies, 236 articles were retained for full-text evaluation.

**Fig. 1:**
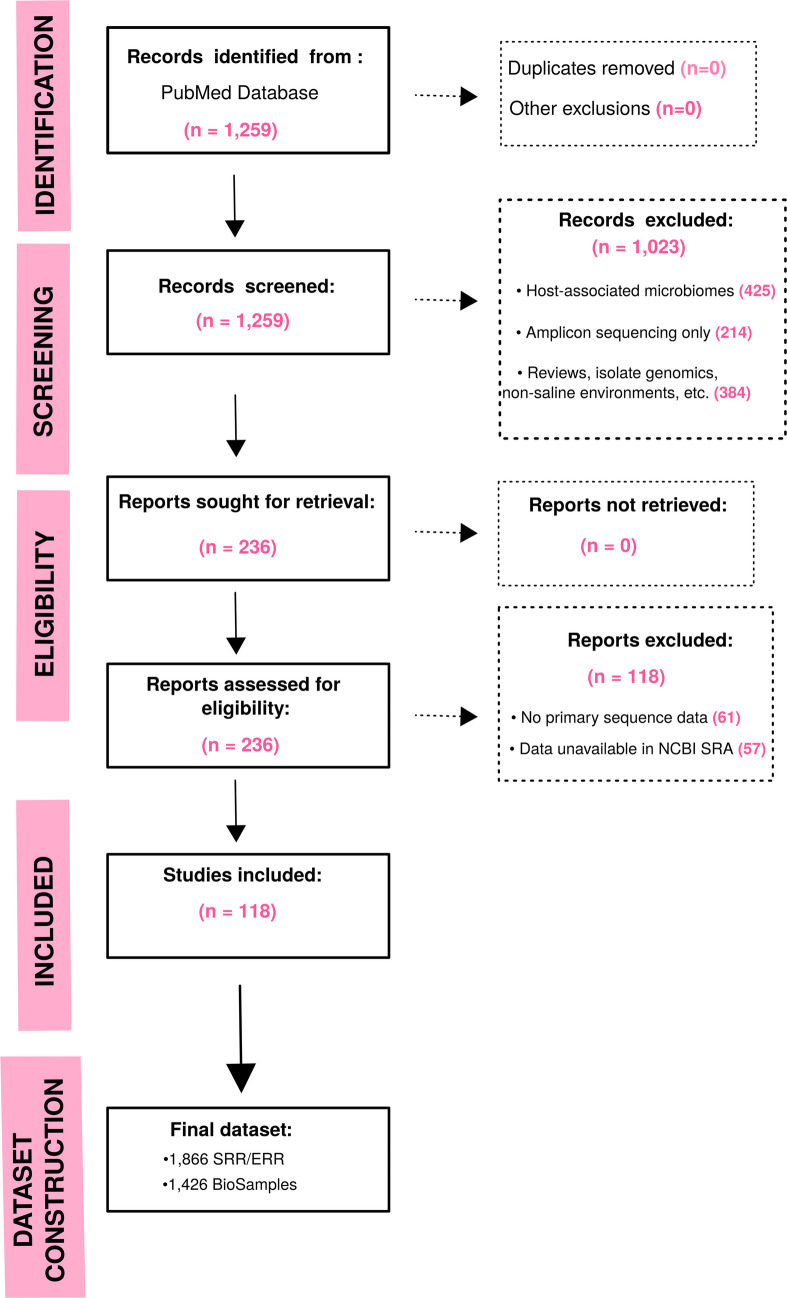
Workflow illustrating the identification, screening, eligibility assessment, and dataset construction steps used to compile the global saline metagenomic dataset. A total of 1,259 records were retrieved from PubMed and manually curated through successive exclusion steps. Ultimately, 118 studies were retained, yielding 2,194 sequence runs (SRR/ERR), of which 1,866 samples corresponding to 1,426 unique BioSamples were included in the final dataset after excluding runs lacking geographic coordinates

Of these, 175 provided primary sequence data, and 118 had their datasets properly deposited in the NCBI SRA database. These 118 articles collectively yielded 2,194 sequence runs (SRR/ERR). Sequencing runs lacking geographic coordinates in their metadata were subsequently excluded. Ultimately, the final vetted dataset comprised 1,866 SRR/ERR samples corresponding to 1,426 unique BioSamples (Fig. 1).

Our literature-first approach carries methodological limitations. By prioritizing high sensitivity in the initial PubMed query to capture studies from underrepresented saline environments, we relied extensively on manual curation. Although this process was conducted rigorously, manual screening inevitably introduces the possibility of researcher fatigue and subjective decisions during title and abstract assessment. In addition, because our workflow was anchored to PubMed-indexed publications to ensure reliable metadata retrieval and traceability, the resulting dataset excludes "orphan" metagenomes deposited directly in public repositories without an associated peer-reviewed article. Consequently, our analysis reflects the global landscape of published halophilic metagenomic studies rather than the complete universe of sequence data currently available in public databases.

### Regional biases in metagenomic data from saline habitats

**Fig. 2:**
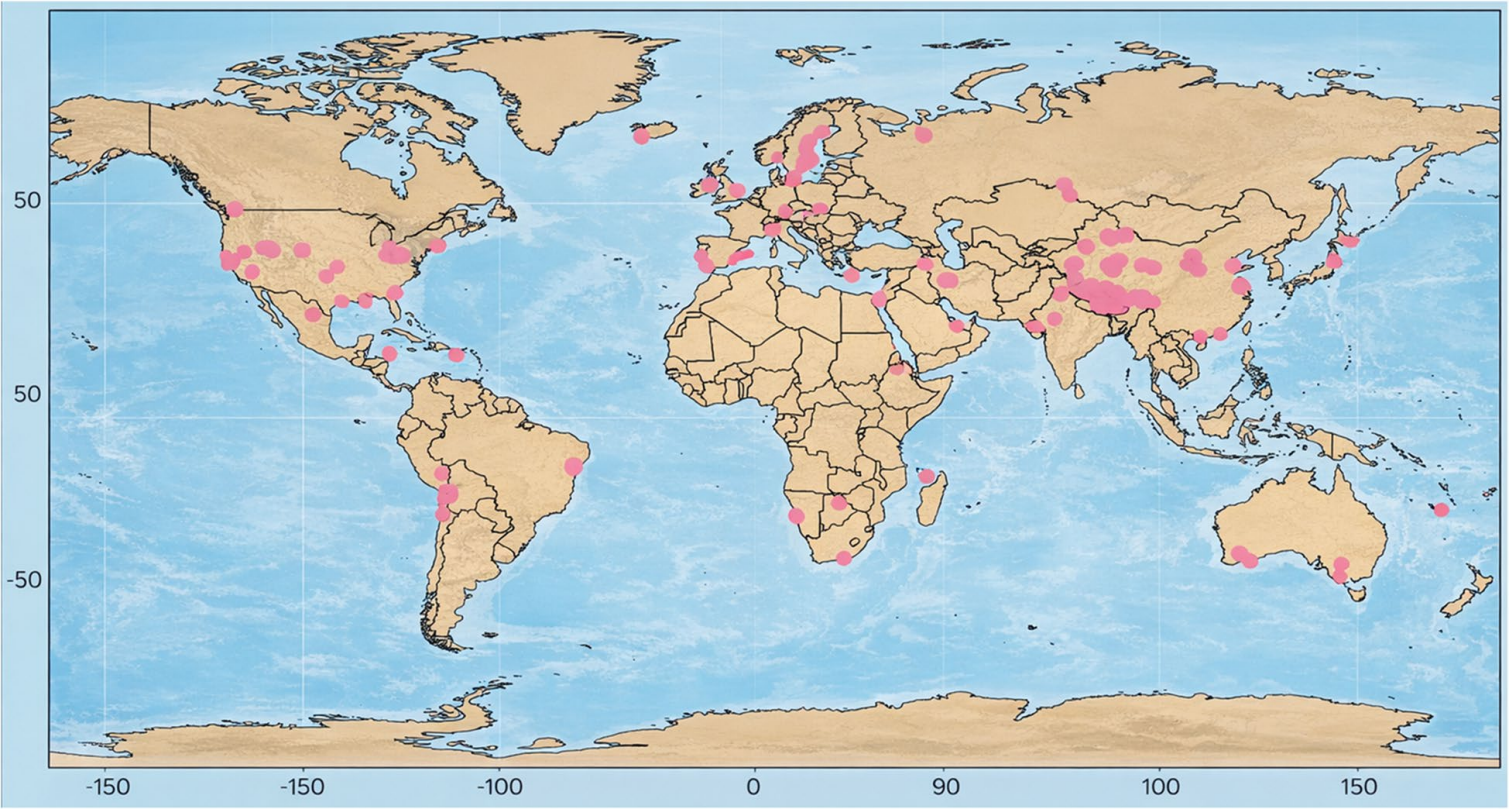
Global distribution of halophilic metagenomic samples available in the NCBI SRA on September 2025

**Fig. 3:**
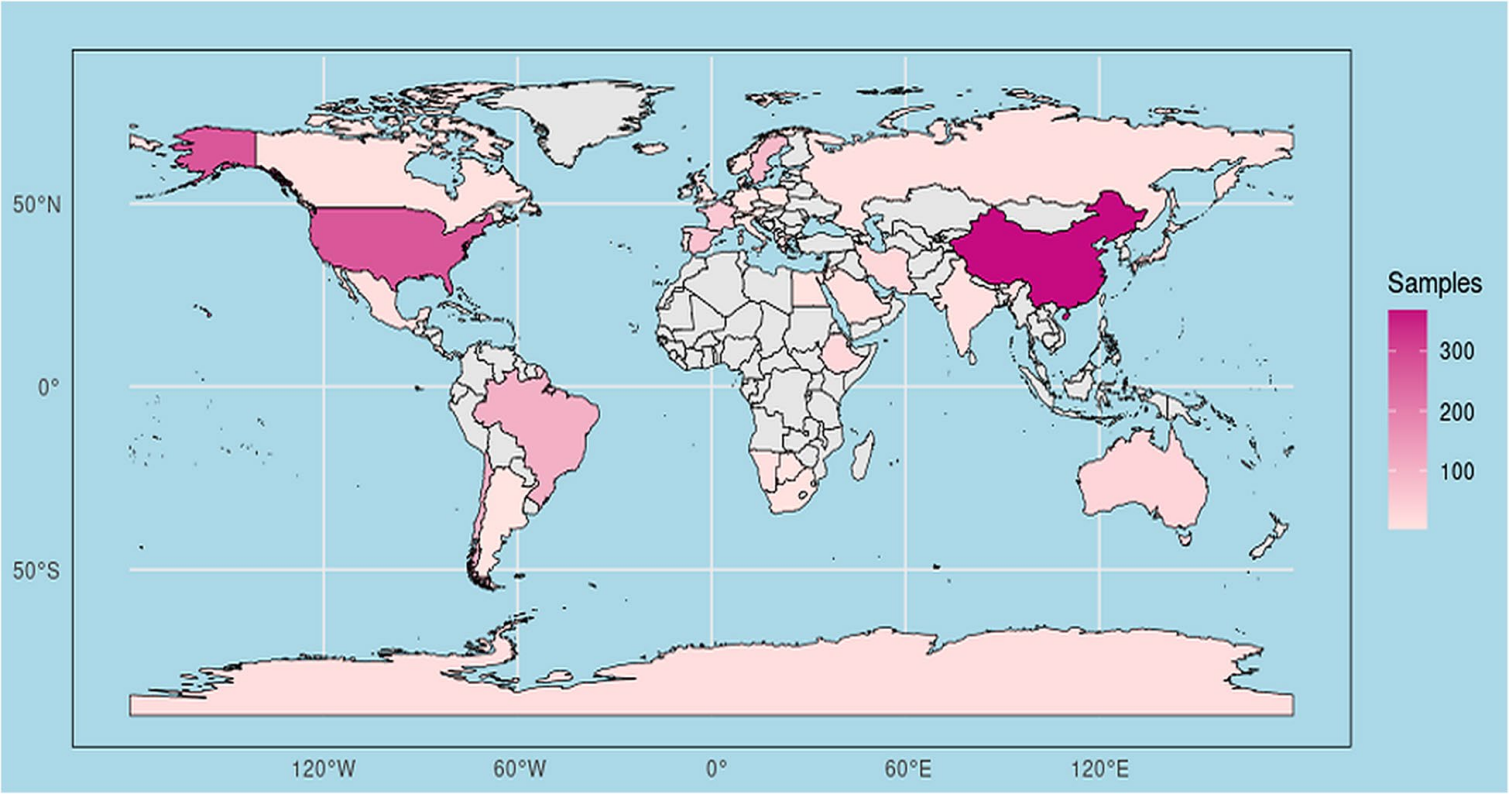
Global distribution of metagenomic samples across countries. Note: Color intensity represents the number of samples available per country, revealing spatial biases in global data coverage

We analyzed metadata from 1,426 NCBI BioSamples originating from 42 countries (Fig. 2). Quantitative analysis of this spatial distribution confirmed a severe inequality in global data availability, yielding a Gini coefficient of 0.736, which mathematically indicates extreme geographic disparity. China and the United States accounted for the largest shares, with 370 (25.94%) and 273 samples (19.14%), respectively (Fig. 3). Together with Europe, which contributed 292 samples (20.48%), these regions dominate the publicly available datasets. Indeed, the top five contributing countries alone account for 65.78% of the entire global dataset. While these countries harbor diverse saline environments, their disproportionate representation more closely reflects sustained investment in scientific infrastructure, sequencing capacity, and data management rather than the true global distribution of hypersaline habitats (Castro Torres and Alburez-Gutierrez 2022). In this context, China’s rapid rise mirrors its substantial expansion in research funding and large-scale sequencing initiatives over the past decade, whereas the long-standing dominance of the United States and Europe aligns with their established research ecosystems and access to advanced genomic technologies (Nguyen and Choung 2020).

Beyond these major contributors, only two additional countries, Chile and Brazil, exceeded 100 samples, and in both cases, sampling was largely driven by geographically concentrated, project-specific efforts. In Chile, most datasets originated from the Atacama Desert, a globally recognized hyper-arid and hypersaline system that has become a reference site for extremophile research and astrobiological analog studies (Azua-Bustos et al. 2022). In Brazil, the majority of samples derived from a single large-scale investigation of mangrove ecosystems in Baía de Todos os Santos (De Carvalho et al. 2024). These examples illustrate how focused national or international initiatives can generate local peaks in data availability without necessarily reflecting broader regional research coverage.

In contrast, Latin America as a whole remains markedly underrepresented, despite hosting a wide diversity of saline and hypersaline environments. Recent syntheses have catalogued numerous extreme systems across the region, including salt lakes, salterns, lagoons, and salt mines, many of which remain minimally explored using metagenomic approaches (Bolivar-Torres et al. 2025). A similar pattern was observed for the Arabian Peninsula, which contributed only 18 samples to our dataset despite encompassing extensive evaporitic salt flats and some of the most chemically and thermally extreme deep-sea brines on Earth, particularly in the Red Sea (He et al. 2024). Together, these patterns indicate that the global landscape of halophilic metagenomic data is shaped less by environmental opportunity than by economic capacity, research infrastructure, and database deposition practices, resulting in substantial geographic blind spots in regions that are highly relevant for both biotechnology and astrobiology.

The underrepresentation of several regions is partly influenced by the methodological and infrastructural filters applied in this study. Our analysis was restricted to publicly available shotgun metagenomes deposited in the NCBI SRA, a sequencing strategy that remains more recent, technically demanding, and financially costly than amplicon-based approaches. As a consequence, research efforts from regions with limited access to high-throughput sequencing or bioinformatics infrastructure are less likely to be captured. In addition, the choice of data repository introduces further bias: for instance, although multiple Brazilian studies on saline environments were identified, only a subset had deposited their datasets in NCBI, with others relying on alternative or less standardized platforms.

Despite these constraints, the magnitude and consistency of the observed geographic disparities suggest that economic factors remain the dominant driver shaping global patterns of metagenomic research in saline ecosystems. Investment in sequencing capacity, data management, and long-term research programs strongly conditions which environments are sampled, analyzed, and ultimately made visible through public databases. This pattern is particularly evident for saline ecosystems, which, beyond their intrinsic ecological and astrobiological relevance, represent valuable reservoirs of biotechnologically relevant enzymes and biomolecules. The disproportionate focus on these environments by countries with greater economic and technological capacity underscores the tight coupling between scientific advancement, resource availability, and the global visibility of microbial diversity.

### Environmental context

Interestingly, the environmental distribution of these samples was less severely skewed than the geographic distribution. While most metagenomic samples derived from a limited set of environmental categories, primarily lakes, sediments, brines, subsurface environments, and soils (Table 1), the overall environmental sampling effort showed moderate uniformity, as mathematically quantified by a high Pielou’s Evenness index (J′ = 0.818). This relative ecological balance contrasts sharply with the extreme geographic disparity observed across nations (Gini coefficient = 0.736), indicating that while public data generation is highly concentrated within a few global territories, the resulting datasets fortunately span a well-distributed range of saline matrices.

**Table 1:** Distribution of samples across environmental contexts

Evironment	BioSamples
Lake	355
Mangrove	40
Marine	109
Mediterranean Forests	10
Sediment	172
Soil	112
Stromatolite	4
Subsurface	120
Surface	12
Antartic Ice	2
Aquatic	15
Arid	48
Beach	6
Brine	158
Desert	15
Estuary	33
Freshwater	15
Groundwater	26
Halite	76
Spring	77
NA	41

The top five categories comprised 897 BioSamples, representing 66.1% of the total dataset. Aquatic systems collectively dominated the available data, contributing 60.2% of all samples. These systems were defined by grouping fluid water-associated environments, specifically lakes, brines, marine waters, springs, estuaries, groundwaters, and unclassified aquatic or freshwaters. This dominance reflects both their ecological prominence and the relative accessibility of water-associated saline environments for sampling and large-scale sequencing efforts. We explicitly note that this distribution reflects historical repository deposition biases and ease of access rather than the true global geographic footprint of these ecosystems.

Many of the sampled habitats are characterized by overlapping extreme conditions that extend beyond hypersalinity alone, underscoring the intrinsically polyextremophilic nature of halophilic microbiomes. Organisms inhabiting these systems are routinely exposed to multiple stressors, including temperature extremes, high hydrostatic pressure, desiccation, nutrient limitation, light deprivation, and elevated ultraviolet radiation. While our metadata-driven analysis does not directly test the functional or taxonomic traits of these microbiomes, the high proportion of datasets originating from these polyextreme settings highlights a strong sampling interest in potential polyextremophilic adaptations. Adaptation to such compound stresses is known to select for metabolic and structural traits that confer exceptional biochemical stability, making these sampled environments a prime target for bioprospecting robust enzymes capable of functioning under harsh industrial conditions (Kumari et al. 2024).

Beyond their biotechnological relevance, these environments also serve as key terrestrial analogs for extraterrestrial settings. Saline springs and subsurface brines, for example, resemble physicochemical conditions hypothesized for the subsurface oceans of Europa and Enceladus, where hydrothermal activity may support microbial life (Weber et al. 2023). Likewise, sites combining high salinity, low temperatures, and intense UV exposure, such as the Antarctic ice, desert, and halite environments represented in our dataset, parallel surface and near-surface conditions on Mars (Godin et al. 2021). Together, these parallels suggest that studying halophilic communities across diverse environmental contexts can inform not only Earth-based applications but also our understanding of the limits of life under extreme conditions and the assessment of habitability within the solar system.

### Temporal trends in halophilic metagenome studies

**Fig. 4:**
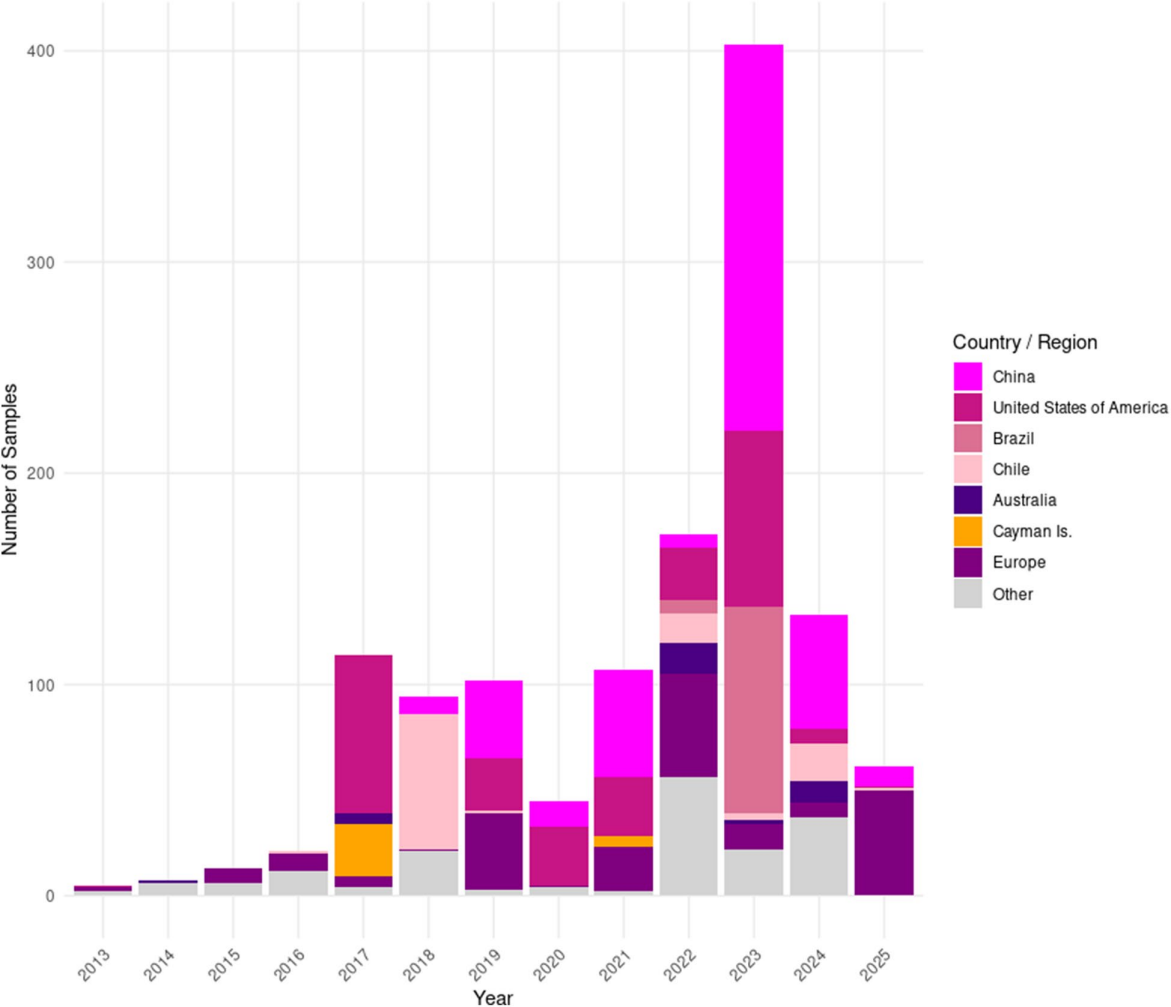
Temporal distribution of samples per country/region

The adoption of shotgun metagenomics has increased over time, driven by advances in sequencing technologies, improved computational pipelines, and a gradual reduction in costs (Liu et al. 2025). Nevertheless, the temporal distribution of halophilic metagenomic datasets does not follow a simple linear growth pattern (Fig. 4). For instance, we observed a pronounced decline in 2020, which aligns temporally with the global disruptions associated with the COVID-19 pandemic. However, because NCBI BioSample release and repository deposition dates inherently lag behind actual field sampling timelines, this drop should be interpreted cautiously as a temporal association within public databases rather than a direct, real-time reflection of interrupted fieldwork. Similarly, the lower number of samples observed for 2024 and 2025 likely reflects these same delays inherent to the publication and data deposition process, as studies conducted during these years may not yet be publicly available.

Conversely, sudden surges in data deposition further distort this timeline. Directly reflecting the geographically concentrated efforts described previously, the temporal peak recorded in 2023 (403 samples) was driven by a handful of large-scale projects rather than a broad expansion of global sampling. Nearly half of the samples generated in 2023 originated from China (183 samples; 45.41%), comprising 169 samples from a single project (Feng et al. 2024), while an additional 98 samples (24.32%) derived from two Brazilian mangrove studies (De Carvalho et al. 2024; Loiola et al. 2023), and 83 samples (20.59%) from the United States, primarily 77 samples from a study focused on the genus *Methanohalophilus* (Borton et al. 2018). Beyond these isolated single-year fluctuations, a broader analysis reveals that China, the United States, and Europe maintain the most persistent presence in data generation over time. This longitudinal stability reiterates that the core output of halophilic metagenomics remains concentrated within the same established research ecosystems.

**Fig. 5:**
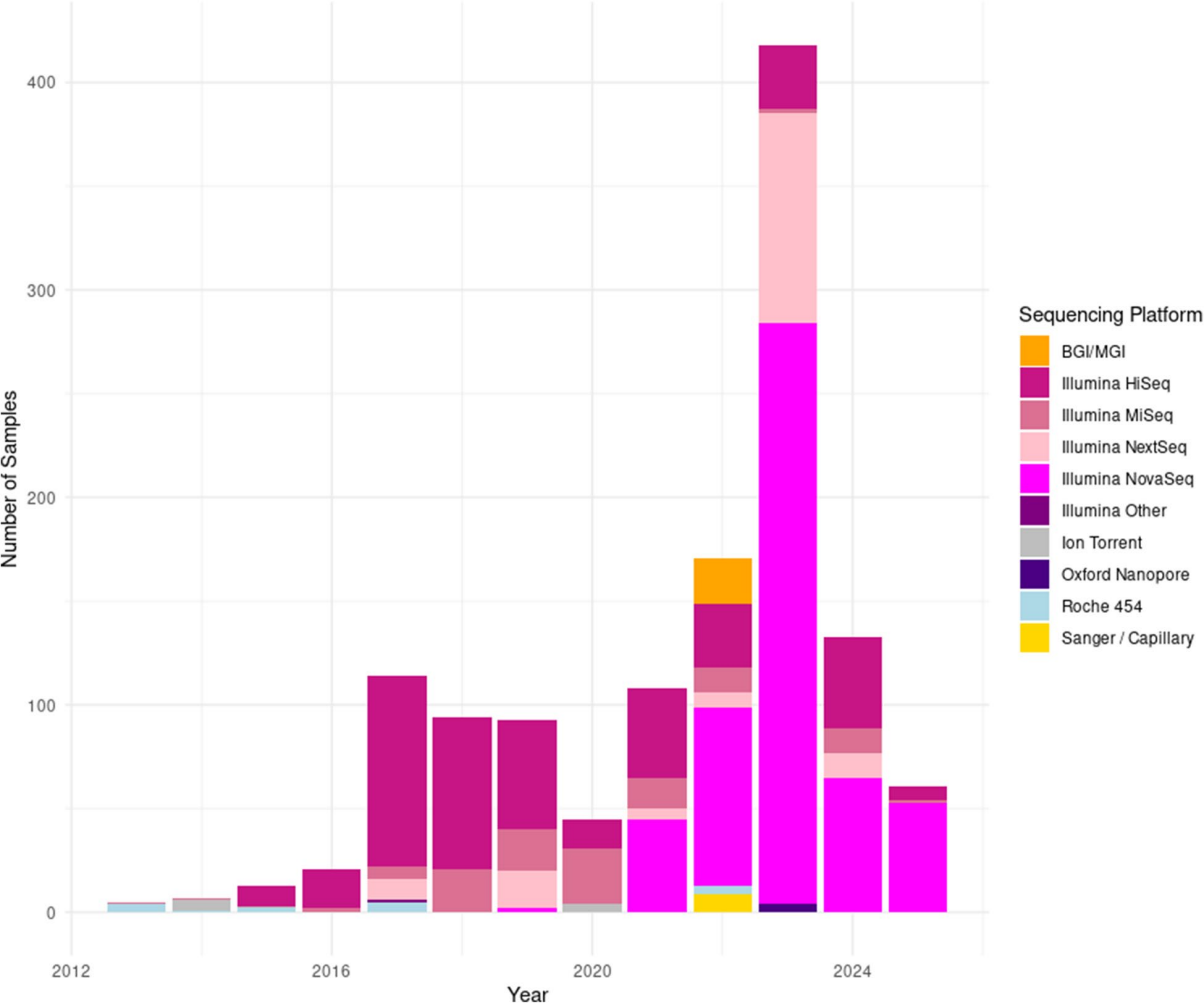
Temporal distribution of samples according to sequencing platform technology

The variation in sample volumes across the timeline is also fundamentally intertwined with the shifting throughput capacity of the sequencing platforms used. Consistent with broader trends in microbial genomics, most datasets in this study were generated using short-read sequencing technologies (Fig. 5). Early datasets primarily relied on pyrosequencing (454 sequencing), which was gradually replaced by Illumina platforms as sequencing costs decreased and throughput increased. Illumina HiSeq 2000 and 2500 dominated between 2017 and 2019, whereas Illumina NovaSeq 6000 became the prevailing platform from 2021 onward. These shifts represent temporal associations with technology access and changing project scales across different regions, rather than an isolated driver of dataset volume. Although NovaSeq was commercially introduced several years earlier, its widespread adoption in this dataset coincides with the typical lag between sample collection, sequencing, and publication, alongside the general workflow slowdowns of the early 2020s. In contrast, Illumina MiSeq exhibited stable usage across multiple years, likely reflecting its accessibility and suitability for small- to medium-scale projects, which broadens regional capacity for baseline environmental monitoring.

## Conclusions and prospects

Our analysis indicates that the global distribution of halophilic metagenomic datasets is shaped less by the availability of saline environments than by disparities in economic investment, research infrastructure, and data deposition practices. Publicly accessible data are dominated by a small number of regions, most notably the United States, China, and Europe, whereas large areas of the world with extensive and diverse saline ecosystems, including much of Latin America and the Arabian Peninsula, remain markedly underrepresented. Chile emerges as a notable exception, as its extreme environments, particularly the Atacama Desert, are already deeply recognized scientifically, culturally, and through tourism, and have consequently supported sustained, environment-focused research initiatives that elevate the country’s visibility in extremophile research beyond broader continental trends.

In addition to geographic imbalance, we identified structural constraints related to repository choice and incomplete metadata reporting, which collectively limit data discoverability, interoperability, and reuse. Although community-driven standards for metadata reporting are readily available, their uneven adoption continues to hinder large-scale comparative analyses of halophilic and polyextremophilic microbiomes. These findings suggest that data fragmentation is not merely a technical limitation but a systemic issue closely linked to funding priorities, infrastructure availability, and misaligned incentives within the global research ecosystem.

Looking forward, addressing these challenges will require coordinated efforts operating at multiple scales. While global interoperability among repositories is essential, our results also highlight the strategic importance of national and regional microbiome initiatives as complementary mechanisms to reduce these systemic inequities. Local frameworks, such as the Brazilian Microbiome Project (Pylro et al. 2014; Pylro et al. 2014), provide robust models that could support halophilic and environmental microbiome research by organizing targeted sampling efforts, standardizing metadata collection, centralizing infrastructure, and expanding regional training in sequencing and bioinformatics. By strengthening local leadership and shared computational capabilities, these initiatives can promote more equitable participation, lower technical barriers for research groups in underrepresented regions, and enable efficient coordination with international consortiums while minimizing duplication and optimizing resource allocation.

Together, strengthened local initiatives and globally interoperable data infrastructures provide a pragmatic pathway toward more representative, efficient, and sustainable halophilic metagenomic research. Broader investment in data generation and analytical capacity, particularly in regions rich in extreme environments but limited in resources, would help ensure that future discoveries in biotechnology and astrobiology reflect the true global diversity of saline ecosystems and the microorganisms they harbour.

## Data Availability

All codes and spreadsheets generated in this study are provided in https://github.com/camilavieira1-byte/Halophilic-Metagenomic-Data-.
